# Dislocation of the Head of the Femur into the Obturator Foramen, in a Child Two Years and Three Months Old. Reduced by Dr. Reid’s Method

**Published:** 1859-03

**Authors:** Chas. A. Bowen

**Affiliations:** St. Catharines, C. W.


ART. III.—*Dislocation of the Head of the Femur into the Obturator Foramen, in a Child two years and three months old. Reduced by Dr. Reid's Method.* By CHAS. A. BOWEN, M. D., St. Catharines, C. W.

Mrs. J., on the 3d of December, 1858, being in a bad humor with things in general, and her child in particular, a little girl two years and three months old, saw fit to pull it violently from where it was sitting, on the floor, by its right leg, and then give it a severe application with her hand, *a posteriori*. As the baby did not cease crying the moment she left off beating it, (which all sensible babies ought to do, no matter how hard or many the slaps,) she caught it by the shoulders. and thumped its gluteal region violently against the seat of a wooden chair, with much force. About half an hour after this *interesting* proceeding I was called to see the child, and found the little sufferer on a woman's lap, moaning most piteously; I asked the woman what was the matter, to which she replied, that the mother had broken one of the baby's legs, as it was longer than the other, and she could not stand on it. After making a careful examination of both inferior extremities, I satisfied myself that the head of the right os femoris was resting in the obturator foramen. The knee was bent, but capable of extension, and when I placed the two legs side by side, the right was about three-fourths of an inch the longer. The foot pointed forwards, and the toes could be inverted or everted, apparently without much pain. The usual flattening of the hips was present, and as the child was rather thin, I could feel the empty acetabulum through the super-imposed tissues. There being no doubt as to the position of the head of the femur, I proceeded to reduce the dislocation, after Dr. Reid's method. Placing the little subject on a table, (the pelvis being steadied by an assistant,) I put my left hand over the seat of dislocation, and grasped the knee with my right, carrying it up and across the opposite thigh, as high as the umbilicus. The knee was next pressed towards the abdomen, to throw the head of the femur out of the thyroid notch, and then shoving up with my left hand, I carried the knee somewhat to the right side, and suddenly straightened the leg. The first and second attempts were alike unsuccessful, but on trying the third time, and using a little more force, I had the satisfaction to feel the head of the bone slip in with a palpable jerk. The child would not put its foot to the floor for the first two weeks, nor did it commence to walk until four weeks had elapsed, and then only a few steps at a time.

It is now eight weeks since the accident, and the little one is running around as well as ever.

